# Which cause of diffuse peritonitis is the deadliest in the tropics? A retrospective analysis of 305 cases from the South-West Region of Cameroon

**DOI:** 10.1186/s13017-016-0070-9

**Published:** 2016-04-11

**Authors:** Alain Chichom-Mefire, Tabe Alain Fon, Marcelin Ngowe-Ngowe

**Affiliations:** Department of Surgery, Faculty of Health Sciences, University of Buea and Regional Hospital Limbe, P.O. Box 25526, Yaoundé, Cameroon

**Keywords:** Diffuse peritonitis, Morbidity, Mortality, Menheim Peritonitis index, Hollow viscus perforation, Septic shock

## Abstract

**Background:**

Acute diffuse peritonitis is a common surgical emergency worldwide and a major contributor to non-trauma related death toll. Its causes vary widely and are correlated with mortality. Community acquired peritonitis seems to play a major role and is frequently related to hollow viscus perforation. Data on the outcome of peritonitis in the tropics are scarce. The aim of this study is to analyze the impact of tropic latitude causes of diffuse peritonitis on morbidity and mortality.

**Methods:**

We retrospectively reviewed the records of 305 patients operated on for a diffuse peritonitis in two regional hospitals in the South-West Region of Cameroon over a 7 years period. The contributions of various causes of peritonitis to morbidity and mortality were analyzed.

**Results:**

The diagnosis of diffuse peritonitis was suggested on clinical ground only in more than 93 % of cases. The most common causes of diffuse peritonitis included peptic ulcer perforation (*n* = 69), complications of acute appendicitis (*n* = 53) and spontaneous perforations of the terminal ileum (*n* = 43). A total of 142 complications were recorded in 96 patients (31.5 % complication rate). The most common complications included wound dehiscence, sepsis, prolonged paralytic ileus and multi-organ failure. Patients with typhoid perforation of the terminal ileum carried a significantly higher risk of developing a complication (*p* = 0.002). The overall mortality rate was 15.1 %. The most common cause of death was septic shock. Differential analysis of mortality of various causes of peritonitis indicated that the highest contributors to death toll were typhoid perforation of terminal ileum (34.7 % of deaths), post-operative peritonitis (19.5 %) and peptic ulcer perforation (15.2 %).

**Conclusion:**

The diagnosis of diffuse peritonitis can still rely on clinical assessment alone in the absence of sophisticated imaging tools. Peptic ulcer and typhoid perforations are still major contributors to death toll. Patients presenting with these conditions require specific attention and prevention policies must be reinforced.

## Background

Pathological conditions requiring surgery contribute significantly to the global disease burden [1]. It is well established that injuries contribute more than 70 % of death toll in the emergency departments of low and middle-income countries (LMICs) [2]. However, non-trauma related conditions are still responsible for a high number of in-hospital deaths and require specific attention, especially in the tropics [2–4].

Acute generalized peritonitis is a common surgical emergency worldwide and has been reported as one of the major contributors to non-trauma deaths in the emergency department despite improvements in diagnosis, surgical treatment and intensive care support [4–6]. The causes of generalized peritonitis vary widely from one setting to another and seem to be correlated to mortality [3, 7, 8]. It is known that community acquired peritonitis represent the vast majority of cases and is largely related to bowel perforation [3, 9]. This latter cause of peritonitis seems to carry the highest mortality rate (10 to 32 %) [7, 9–12]. Analysis of the contribution of various forms of perforative peritonitis to morbidity and mortality indicate that while results of treatment of peritonitis secondary to peptic ulcer perforation seem to have improved over the past decades [13–15], other frequent causes in the tropics such as typhoid fever related perforation of the small bowel still carry a heavy morbidity and mortality rates [4, 16–18].

Some factors influencing outcome of peritonitis which have been studied and reported so far include age, co-morbidities, severity of sepsis, delay before initiation of treatment and immune suppression [3, 6, 8]. Early prognostic evaluation of patients with acute generalized peritonitis is desirable to select patients with a higher risk of adverse event who may be eligible for a more aggressive treatment. Various approaches to anticipate the outcome by grading the severity of peritonitis have been proposed. They generally rely on scoring systems such as APACHE II and the Mannheim Peritonitis Index (MPI).

Data on the burden and outcome of peritonitis in sub-Saharan Africa are very scarce and few studies have attempted a differential analysis of various causes of diffuse peritonitis. As a consequence, surgeons performing in these areas of the world generally lack management guidelines which are adapted to their local conditions characterized by absence of health insurance, poor technical background and limited access to intensive care unit.

The aim of this study is to identify the most common causes of diffuse peritonitis in the tropical latitudes and their relative contribution to morbidity and to death toll. The ultimate goal is to help surgeons identify cases which are likely to require a more aggressive therapy and rationalize the decision to refer patients towards a center with an intensive care unit. We hypothesized that peritonitis secondary to peptic ulcer perforation was the highest contributor to death toll in the tropics.

## Methods

### Study design and setting

This observational retrospective analysis covered a period of 7 years (from January 01^st^ 2007 to December 31^st^ 2013) in the two regional hospitals of the Fako division in the South-West Region of Cameroon. These level III institutions are located in the cities of Limbe and Buea respectively and are easily accessible from most tributary health institutions thanks to the acceptable road network of the Fako division. They have a total admission capacity of 326 beds. The total catchment population is estimated at 527,000 people. These two institutions are organized in a similar model with an emergency department where all urgent cases are initially admitted. Cases requiring surgery are transferred to corresponding surgical wards with a cumulated admission capacity of 58 beds managed by four surgeons during the study period. Surgical interventions are carried out in one of the two operative rooms of each institution. They both possess a laboratory and an imaging department where most basic work-up can be performed. Computerized tomography, bacterial culture and intensive care units are available in none of the institutions. Cases requiring more specialized investigations or intensive care can however be referred to the city of Douala located about 70 km from both cities where two large central hospitals possessing all the services are available and functional.

### Study population and procedure

We included in this study all patients operated on for an intra-abdominal sepsis for which a final diagnosis of diffuse peritonitis was made. Diffuse peritonitis was defined as any intra-abdominal infection extending beyond the transverse mesocolon. The exclusion criteria were the following:

- All patients with a localized peritonitis.
- All patients with a primary peritonitis defined as diffuse peritonitis with no identifiable source of infection during surgical exploration.
- All patients with suspected peritonitis for whom a laparotomy was not performed.
- All patients whose file did not contain follow-up data.

Data source included admission registers of the emergency department, patient’s admission files, post-operative note registers and report books of the surgical wards. For each patient included, we recorded on a pre-designed data collection form data regarding patient’s characteristics, clinical and para-clinical characteristics of the peritonitis, findings of the surgical exploration, follow-up data and final outcome. Sepsis, septic shock and multiorgan failure were defined according to the American College of Chest Physicians/Society of Critical Care Medicine Consensus Conference Committee of 1991 as modified in 2001 [19, 20]. Only adverse events occurring during the same admission were considered.

The characteristics of the peritonitis were classified according to the MPI which has been extensively used to predict the outcome of various forms of peritonitis [6, 21, 22]. The severity of complications was graded according to the Clavien-Dindo classification [23, 24].

### Statistical analysis

All data were entered in an excel database (Excel 2007, Microsoft corporation®) and later one converted into an Epi-info 7 for the purpose of statistical analysis. Pairwise comparisons were done using Epi-info Statalc function. Spontaneous comparisons were done using STATA 10.

### Ethical consideration

The procedures of this study respected the Helsinki declaration and were in conformity with the laws of the republic of Cameroon about research on human subjects. An ethical approval was obtained from the Institutional Review Board of the University of Buea.

### Reporting

The STROBE guidelines were used in reporting this study [25].

## Results

### Patient’s characteristics

A total of 378 patients were admitted in these two institutions with the post-operative diagnosis of acute diffuse peritonitis over the study period.

These included 230 patients from Buea Regional Hospital and 148 patients from Limbe Regional hospital. A total of 73 patients were excluded for the following reasons:

- Thirty four records had incomplete data. These included four patients with a presumptive diagnosis of peritonitis who died before a laparotomy could be performed.
- For the remaining 39 files, analysis of the operative notes indicated that no cause was identified for the peritonitis during surgical exploration and they were classified as primary peritonitis.

A total of 305 files could finally be analyzed, 201 (65.9 %) from Buea Regional Hospital and 104 (34.1 %) from Limbe Regional Hospital.

**Fig. 1:**
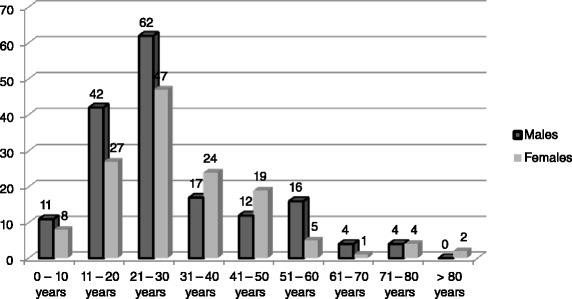
Age and sex distribution of cases of diffuse peritonitis in Limbe and Buea Regional Hospitals

Our sample included 168 males and 137 females, giving a sex-ratio of 1.23/1. The ages of our patients ranged from 3 to 82 years with a mean of 30.6 ± 16.0 years. As shown on Fig. 1, a total of 269 patients (88.2 %) were aged 50 years or below.

### Characteristics of the peritonitis

As shown in Table 1, the most common clinical findings were diffuse abdominal pain (100 %), abnormal temperature (83 %) and signs of peritoneal irritation (tenderness, rebound tenderness, guarding, rigidity: 91 %). A total of 138 patients (45 %) presented with signs of sepsis on admission. The delay between onset of symptoms and admission ranged from 16 h to 9 days with a mean of 3.62 days.

**Table 1:** Clinical and para-clinical characterisitics of diffuse community acquired peritonitis in Limbe and Buea Regioanl Hospitals

Clinical and para-clinical findings	Number	Percentage
Abdominal pain	305	100
Nausea/vomiting	128	42
Diarrhea/constipation	214	70.1
Fever or hypothermia	253	83
Tachycardia	219	71.8
Tachypnoea	133	43.6
Abdominal distention	198	64.7
Signs of peritoneal irritation	277	90.8
Signs of shock	138	45.1
Leucocyte count >12.000	138/196	80.4
Leucocyte count < 4000	32/196	16.3
Pneumoperitoneum	86/231	37.22
Air fluid levels	82/231	35.5
Suggestive ultrasound findings	156/238	96.9

Most patients (80 %) for whom a leucocyte count was requested and had a leucocytosis above 12.000/ml.

The diagnosis of acute generalized peritonitis was suspected on clinical ground in all cases and the most common confirmatory tool was ultrasound used in 238 (78 %) cases. The cause of peritonitis was suspected pre-operatively based on the combination of clinical and ultrasonographical findings in 246 (81 %) of cases. An erect chest X-ray was requested and performed in 231 (75.7 %) patients and revealed a pneumoperitoneum in 37 % of cases, all with a final diagnosis of either peptic ulcer or small bowel perforation.

**Fig. 2:**
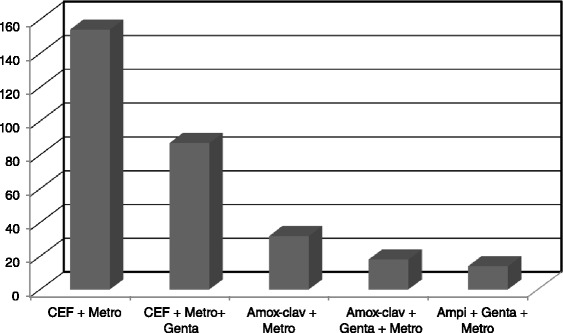
The various antibiotic regimens proposed to patients with diffuse community acquired peritonitis in Limbe and Buea. CEF + Metro: combination of ceftriaxone and metronidazole. CEF + Metro + Genta: combination of ceftriaxone, metronidazole and gentamicine. Amox-clav + Metro: combination of amoxicillin-clavulanic acid and metronidazole. Amox-clav + Metro + genta: combination of amoxicillin-clavulanic acid, metronidazole and gentamicine. Ampi + Genta + Metro: combination of Ampicillin,Gentamicine and Metronidazole

All patients with a suspicion of diffuse peritonitis had an antibiotic regimen started in the emergency department. As shown on Fig. 2, most patients (79 %) received a combination of ceftriaxone and metronidazole with or without gentamicine.

Table 2 indicates all the causes of diffuse peritonitis as reported by the surgical exploration. According to this table, the five most common causes included peptic ulcer perforation (*n* = 69), complications of acute appendicitis (*n* = 53), post-operative peritonitis (*n* = 44), typhoid related perforation of the terminal ileum (*n* = 43) and abdominal injuries (38). As Table 3 shows, the age distribution of these five most common causes of diffuse peritonitis indicates that almost 75 % of cases of typhoid perforation of small bowel occurred before the age of 20. Also, 26 of the 44 cases of post-operative diffuse peritonitis (59 %) were consecutive to the septic complications of illegal abortion, performed by a health care provider out of the hospital in most cases.

**Table 2:** Relative frequency and sex distribution of causes of diffuse community acquired peritonitis in Limbe and Buea Regional hospitals

Cause	Males	Females	Total	Percentage
Peptic Ulcer Perforation	49	20	69	22.6
Spontaneous perforation of terminal ileum	19	24	43	14.1
Complications of acute appendicitis	34	19	53	17.4
Splenic Abscess	4	2	6	2
Tubo-Ovarian Abscess	0	7	7	2.3
Acute cholecystitis	1	7	8	2.6
Incarcerated hernia	8	0	8	2.6
Intestinal obstruction	4	9	13	4.3
Intussusception	3	0	3	1
Volvulus of sigmoid colon	8	1	9	3
Infection of haemoperitoneum	0	2	2	0.6
Rupture of liver abscess	1	1	2	0.6
Hospital-acquired	10	34	44	14.4
Blunt abdominal injury	21	6	27	8.9
Penetrating abdominal injury	6	5	11	3.6
Total	168	137	305	100

**Table 3:** Age distribution of the five most common causes of diffuse community acquired peritonitis in Limbe and Buea

Age group	Peptic ulcer perforation	Perforation of terminal ileum	Complications of appendicitis	Hospital-acquired	Abdominal injuries	Total
0–10 years	0	11	3	0	6	20
11–20 years	14	21	14	12	13	74
21–30 years	27	5	22	22	11	87
31–40 years	15	3	10	7	4	39
41–50 years	7	1	3	3	1	15
51–60 years	4	1	1	0	2	8
61–70 years	0	1	0	0	1	2
71–80 years	1	0	0	0	0	1
>80 years	1	0	0	0	0	1
Total	69	43	53	44	38	247

**Fig. 3:**
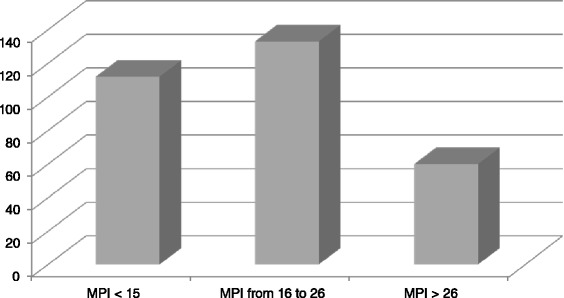
Distribution of cases of diffuse peritonitis in Limbe and Buea Regional Hospitals according to Mannheim Peritonitis index

When assessing the severity of the peritonitis, the MPI ranged from 6 to 34 points with a mean of 19.88 ± 9.68. We divided our patients in three groups: those with a MPI of <15, those with MPI ranging from 16 to 25 and those with MPI >26. As shown on Fig. 3, 60 (19.7 %) patients had a MPI > 26.

Analysis of post-operative notes indicated that source control was successful in 286 patients (93.8 %). All cases of peptic ulcer perforation were located on the proximal duodenum, except for three cases of gastric ulcers. The most frequent treatment modality for cases of peptic ulcer perforation was suture with omentum patch after Graham applied in 92.8 % of patients. Three patients (4.34 %), all from Limbe Regional hospital had a bilateral trunkal vagotomy performed as definitive treatment of the peptic ulcer disease. All typhoid related perforations of the small bowel were located in the last 100 cm of the ileum. Simple suturing of the ileal perforation was the most frequently used treatment modality applied in 31 (74.4 %) of patients.

### Outcome

**Fig. 4:**
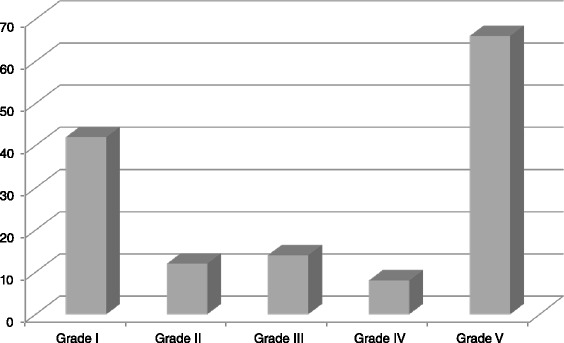
Distribution of complications recorded in our patients according to the Clavien-Dindo classification

The outcome data are shown in Table 4-6. A total of 142 complications were recorded in 96 patients (31.5 % complication rate). The most common complications recorded included wound dehiscence, sepsis, prolonged paralytic ileus and multi-organ failure. The most common combination was the association of signs of septic shock with paralytic ileus. According to the Clavien-Dindo classification, as shown in Fig. 4, when excluding those who died (classified as Clavien-Dindo V), the majority of patients developed a Grade I complication. A total of 100 of these complications occurred in 84 of the 247 patients whose laparotomy was performed for one of the five most common causes of diffuse peritonitis listed above (34 % complication rate). According to Table 7, septic shock and multi-organ failure were very frequent complications in patients with typhoid perforation of the ileum. Patients with MPI of 16 or more carried a significantly higher risk of developing a complication (*P* < 0.0001). Differential analysis indicates that patients with typhoid perforation of the terminal ileum carried a significantly higher risk of developing a complication (*p* = 0.002).

**Table 4:** outcome of the management of diffuse peritonitis in Limbe and Buea Regional Hospitals

Complications recorded		
Type of complication	Number recorded	Percentage
Sepsis	28	9.2
Respiratory infection	6	2
Multi-organ failure	17	5.6
Wound dehiscence	36	11.8
Prolonged paralytic ileus	23	7.5
Post-operative peritonitis	12	4
Post-operative fistula	4	1.3
Residual/recurrent abscess	16	5.2

**Table 5:** Outcome of the management of diffuse peritonitis in Limbe and Buea Regional Hospitals

Complication rates for the five most common causes of diffuse peritonitis					
Cause of peritonitis	Number with complications	Complication rate	Risk ratio (RR)	95 % CI	Fisher’s *P*-value
Peptic ulcer perforation	18	25.4 %	0.77	0.50, 1.19	0.25
Perforation of ileum	25	58.1 %	1.77	1.30, 2.41	0.002
Acute appendicitis	13	24.5 %	0.74	0.45, 1.23	0.26
Post-operative	14	31.8 %	0.97	0.61, 1.54	1.00
Abdominal injury	12	31.6 %	0.96	0.58, 1.58	1.00

**Table 6:** Outcome of the management of diffuse peritonitis in Limbe and Buea Regional Hospitals

Analysis of mortality rate						
Cause of peritonitis	Number of deaths	Mortality rate	Contribution to death toll	Risk ratio (RR)	95 % CI	Fisher’s *P*-value
Peptic Ulcer Perforation	7	10.1 %	15.2 %	0.67	0.32, 1.43	0.34
Spontaneous perforation of terminal ileum	16	37.2 %	34.7 %	2.47	1.54, 3.95	0.001
Complications of acute appendicitis	4	7.6 %	8.7 %	0.50	0.19, 1.33	0.20
Splenic Abscess	0	0	0	0	Undefined	0.60
Tubo-Ovarian Abscess	0	0	0	0	Undefined	0.60
Acute cholecystitis	0	0	0	0	Undefined	0.61
Incarcerated hernia	1	12.5 %	2.2 %	0.83	0.13, 5.29	1.00
Intestinal obstruction	2	15.4 %	4.4 %	1.02	0.28, 3.75	1.00
Intussusception	0	0	0	0	Undefined	1.00
Perforation of sigmoid colon	4	44.4 %	8.7 %	2.95	1.35, 6.41	0.04
Infection of haemoperitoneum	0	0	0	0	Undefined	1.00
Rupture of liver abscess	0	0	0	0	Undefined	1.00
Post-operative	9	20.5 %	19.5 %	1.36	0.71, 2.57	0.38
Abdominal injury	3	7.9 %	6.6 %	0.52	0.17, 1.60	0.33
Total	46	15.1 %	100 %	Ref.	-	**-**

**Table 7:** Complications recorded in patients operated for the five most common causes of peritonitis in Limbe and Buea regional Hospitals

Cause	Peptic ulcer perforation	Perforation of terminal ileum	Complications of appendicitis	Post-operative	Abdominal injuries	Total
Complication						
Septic shock	4	10	2	4	1	21
Respiratory infection	3	1	0	0	1	5
Multi-organ failure	3	5	2	2	0	12
Surgical site infection	1	3	6	0	1	11
Wound dehiscence	4	5	1	0	1	11
Prolonged paralytic ileus	4	6	1	2	2	15
Post-operative peritonitis	2	1	2	4	1	10
Post-operative fistula	0	2	0	0	0	2
Residual abscess	2	3	2	3	3	13
Total	23	36	16	15	10	100

A total of 46 patients were reported death during the course of management, giving an overall mortality rate of 15.1 %. The most common cause of death was septic shock. Those who died each developed a mean of 1.43 complications. Two patients died in the operative room, both with a severe pre-operative sepsis. Differential analysis of mortality of various causes of peritonitis indicated that the highest contributors to death toll were perforation of terminal ileum (34.7 % of deaths), post-operative peritonitis (19.5 %) and peptic ulcer perforation (15.2 %). As shown on Table 4, perforation of sigmoid colon, perforation of the terminal ileum and post-operative peritonitis carried a significantly higher relative risk of death.

## Discussion

This study is one of the few conducted in the LMICs, that includes a large sample size and analyzes complications and fatality rates for various causes of diffuse peritonitis.. It is a contribution to the advocacy in favour of global surgery as outlined by the Lancet commission for Global surgery and its objectives for the year 2030 and by the World Health Assembly’s resolutions on the need to reduce the global burden of surgical conditions potentially correctable by surgery, especially in Low and middle income countries [26, 27].

Our study suggests that spontaneous perforation of small bowel, usually typhoid fever related is a substantial problem especially in paediatric populations. Also, peptic ulcer perforation is still a major concern in these areas of the world. Septic complications of illegal abortions also require a specific attention. Large proportion of patients with diffuse peritonitis still present to the hospital with unacceptable delays and this probably accounts for the high incidence of sepsis and high MPI scores at the time of diagnosis with the consequences that it entails in terms of outcome. In settings with limited technical background, the diagnosis of this common clinical entity can still rely largely on clinical arguments. Patients operated on for diffuse peritonitis are likely to develop wound dehiscence, sepsis, prolonged paralytic ileus or multi-organ failure. These complications often occur in combination especially in those with typhoid related small bowel perforation, and can be deadly in more than 15 % of cases. The highest contributors to death toll are all cases of peritonitis originating from bowel perforations, especially those related to complications of typhoid fever which is endemic in the region.

This study brings to light once more the crucial problem of filing and conservation of data in LMICs with nearly 10 % of patients excluded for incomplete data. However, higher rates of patients with incomplete files have been reported in similar settings [2]. Also, it is questionable how the findings of this study can be compared to those from other centers where all the facilities for diagnosis and management are available. In particular, the absence of equipment for the laparoscopic approach is likely to influence the outcome. It has been reported that this approach could be proposed to as much as 27 % of patients [5] with a supposedly better outcome. Our choice to limit this study to diffuse peritonitis is inspired by the fact that this form of peritonitis is by far the most frequent with a higher death toll [3–5].

While multiple reports indicate that diffuse peritonitis, especially when related to bowel perforation seem to affect young patients with a predominance of male sex [4, 8, 17, 28, 29], major differences in causes between LMICs and developed countries have been reported. In general, patients from LMICs tend to suffer perforations of the proximal gut while does in the western countries are more often affected with perforations of the large intestine [30]. The five most common causes of secondary peritonitis described in our study have been reported in numerous studies in similar settings [7, 9, 12, 29, 31, 32]. Peptic ulcer perforation is still a frequent complication and affects the duodenum in the large majority of cases [7, 9, 33, 34]. Typhoid related perforation of the ileum appears to be a major problem in paediatric populations together with appendicular peritonitis [35–37]. Involvement of the biliary tract is rare as opposed to findings of western countries [5]. Health care induced peritonitis represents a smaller fraction but tend to be more severe [4, 38].

Late presentation is a major concern in many areas of the world and delays as long as 13 days have been reported [11, 16, 17]. The absence of modern diagnostic tools in settings with limited technical background cannot be considered a major problem as diffused peritonitis can generally be diagnosed or at least suspected on purely clinical grounds in more than 97 % of cases [8, 39].

The choice of antibiotics seem to rely to a large extend on the fact that *E. coli* has been identified as the most frequent causative agent [8, 40]. Its sensitivity pattern validates our choice of antibiotics combination which elements are very widely used [40, 41], although some studies have reported other germs with a different sensitivity pattern [42]. The replacement of 3^rd^ generation cephalosporin by ampicillin in the protocol has been proved to be a valid cost-effective regimen, especially if combined with gentamicin [43]. The use of chloramphenicol must be advocated in cases of perforation of terminal ileum suspected to be of typhoid origin [29]. Tertiary peritonitis is frequently polymicrobial and a strategy to tackle fungal infection needs to be considered [3, 38].

Although numerous scoring systems have been proposed to assess the severity of peritonitis, MPI has been largely recognized as a valid and reliable predictor of outcome [6, 8, 21, 44]. This simple, purely clinical assessment tool is particularly adapted to settings with limited access to para-clinical work-up tools and can be extensively used with accuracy comparable to other validated tools such as the various version of the APACHE scoring system [5, 10].

There is strong evidence that the management of diffuse peritonitis should still rely on three fundamental principles: (1) Elimination of the source of infection; (2) reduction of bacterial contamination of the peritoneal cavity; and (3) prevention of persistent or recurrent intra-abdominal infection [4]. Concerning the suppression of the cause, the source of peritonitis can usually be controlled in almost 90 % of cases [4, 28, 45]. Generally it appears that surgeon seem to be generally reluctant using the laparoscopic approach [5]. It has been proven that the results of this approach are equivalent to those of open surgery [13]. In peptic ulcer perforations, the surgical definitive treatment of the peptic ulcer disease is rarely proposed and procedures such has suture and omentoplasty after Graham is generally considered sufficient on the condition that the medical treatment be proposed post-operatively [3, 46]. This approach has the advantage of shortening the operation time and improving the outcome, especially in patients with sepsis. In fact, the results of treatment of all bowel perforation seem to favour simple suturing rather that resections and anatomosis, especially in typhoid related perforations of the small bowel [3, 16, 46–48]. The need to protect the suture or anastomosis with a loop ileostomy has been discussed [36]. The prevention of persistent intra-abdominal infection currently opposes two strategies: on-demand re-laparotomy and systematic planned relaparotomies. Current literature seem to favour the on-demand approach in terms of length of hospitalization and intensive care unit stay [3, 49–51].

Morbidity and mortality rates are extremely variable and do not seem to be superior in settings with a limited technical background [4, 8, 9, 18, 28, 29, 39, 45, 52], even in tertiary peritonitis [38]. The mortality rate reported in our study is unacceptably high. This is probably a direct consequence of some of the local conditions of surgical practice such as the scarcity of surgeons, the lack of appropriate diagnosis and management tools and the socio-economic conditions characterized by the total absence of social security even for such critical and potentially deadly conditions. Also, they are no clear standards and guidelines for the management of surgical emergencies which are adapted our settings. However, this heavy mortality rate is not exceptional. It is comparable to what have been reported in other regions and countries with similar settings [43, 46]. Even in some western countries, overall complication rates as high as 41 % have been reported [39, 45].

In differential analysis of relative contributors to death toll, our study clearly points complications of typhoid fever as a major problem. Over the past two decades, the trend of mortality of this type of peritonitis has been on the decline [16, 53, 54]. Such reduction can only be achieved by early recognition and diagnosis, timely surgical intervention, appropriate antibiotics and surgical technique and peri-operative care which all play a key role in reducing mortality in typhoid intestinal perforation [53]. Also, policies on typhoid vaccine and public health education may help to reduce morbidity and mortality due to this endemic disease [55].

Some factors have been reported as related to the morbidity of diffuse peritonitis. One of these factors is the delay before intervention which is considered by many as an important key [2, 4, 6, 17, 56, 57]. Other factors include the source of peritonitis with a higher complication rate for bowel perforations [4, 52, 58] and MPI [21, 22, 56]. The ability to suppress the source of infection also seems to play an important role [58].

The types of complications recorded in our study are generally the rule, especially in low-income settings [29, 31, 37, 59, 60]. Adesunkanmi et al. recorded 58 % of wound dehiscence in a neighbouring country [29].

Despite all the recent advances in the medical management of peptic ulcer disease, its contribution to the death toll of diffuse peritonitis is still unacceptably high and can be predicted with special scoring systems [14, 15, 34, 61]. It has been reported that the number of deaths attributable to peptic ulcer perforation is seven times the one of acute appendicitis [13]. Although the outcome of management of typhoid related perforation of small bowel seems to have improved over the recent years, it is still frequently reported as a major contributor to mortality rates [1, 18, 47]. Recognized mortality factors include age, origin of sepsis, MPI greater than 26 and multi-organ failure [6, 8, 21, 44, 58, 62]. Demmel et al. reported more than 50 % of sepsis related deaths [21].

## Conclusion

Diffuse peritonitis is still a major life-threatening condition in LMICs. The diagnosis can reasonably still rely to a very large extend on a meticulous clinical assessment rather than sophisticated tools such as CT scan. In all cases, the clinical assessment must lead to the estimation of severity based on simple but reliable grading systems such as the MPI. Peritonitis originating from the perforation of a hollow viscus deserves special attention. The morbidity and mortality rates of diffuse peritonitis in the Fako are unacceptable high and health authorities need to consider the need for financing the management of such life-threatening surgical conditions as it is the main way to mprove their outcome. Some specific situations require special attention based on public health intervention. These include typhoid ileal perforation for which prevention and early detection are desirable, especially in children. Once the peritonitis has occurred, the adjustment of antibiotic regimen to match the special sensitivity pattern of Salmonella typhi will likely improve overall outcome. The same approach is applicable to complications of peptic ulcer perforation for which the reinforcement of the identification and management of patients suffering from this medical condition before perforation occurs would be beneficial.

For the prevention of persistent abdominal sepsis, surgeons in low-income setting can safely apply the on demand re-laparotomy approach which is likely to be cost-effective.
